# Work Systems Analysis of Emergency Nurse Patient Flow Management Using the Systems Engineering Initiative for Patient Safety Model: Applying Findings From a Grounded Theory Study

**DOI:** 10.2196/60176

**Published:** 2024-12-10

**Authors:** Ellen Benjamin, Karen K Giuliano

**Affiliations:** 1 Elaine Marieb College of Nursing, University of Massachusetts Amherst, Amherst, MA, United States; 2 Donna M and Robert J Manning College of Nursing and Health Sciences, University of Massachusetts Boston, 100 Morrissey Blvd, Boston, MA, 02125, United States, 1 6172875000; 3 Elaine Marieb Center for Nursing and Engineering Innovation, Institute for Applied Life Sciences, University of Massachusetts Amherst, Amherst, MA, United States

**Keywords:** patient flow, throughput, emergency department, nursing, emergency nursing, organizing work, cognitive work, human factors, ergonomics, SEIPS model

## Abstract

**Background:**

Emergency nurses actively manage the flow of patients through emergency departments. Patient flow management is complex, cognitively demanding work that shapes the timeliness, efficiency, and safety of patient care. Research exploring nursing patient flow management is limited. A comprehensive analysis of emergency nursing work systems is needed to improve patient flow work processes.

**Objective:**

The aim of this paper is to describe the work system factors that impact emergency nurse patient flow management using the System Engineering Initiative for Patient Safety model.

**Methods:**

This study used grounded theory methodologies. Data were collected through multiple rounds of focus groups and interviews with 27 emergency nurse participants and 64 hours of participant observation across 4 emergency departments between August 2022 and February 2023. Data were analyzed using coding, constant comparative analysis, and memo-writing. Emergent themes were organized according to the first component of the System Engineering Initiative for Patient Safety model, the work system.

**Results:**

Patient flow management is impacted by diverse factors, including personal nursing characteristics; tools and technology; external factors; and the emergency department’s physical and socio-organizational environment. Participants raised concerns about the available technology’s functionality, usability, and accessibility; departmental capacity and layout; resource levels across the health care system; and interdepartmental teamwork. Other noteworthy findings include obscurity and variability across departments’ staff roles titles, functions, and norms; the degree of provider involvement in patient flow management decisions; and management’s enforcement of timing metrics.

**Conclusions:**

There are significant barriers to the work of emergency patient flow management. More research is needed to measure the impact of these human factors on patient flow outcomes. Collaboration between health care administrators, human factors engineers, and nurses is needed to improve emergency nurse work systems.

## Introduction

### Background

Emergency department crowding poses a grave threat to global health [1]. Hospitals face high patient volumes and acuity, limited bed capacity, staffing shortages, and financial constraints [1,2]. These issues necessitate an urgent optimization of patient flow to provide timely, efficient, high-quality care [2,3]. Emergency nurses play a central role in effective patient flow management, but their work is poorly understood [4-6]. This paper describes the human factors that impact the work of emergency nurse patient flow management.

### Patient Flow Management

Patient flow analysis is the study of progressive patient movement through a unit, hospital, and wider health care system [6,7]. Research analyzing patient flow has grown over the last 2 decades to address patient crowding and limited health care resources [1,2]. The study of patient flow is especially important within emergency departments, where overcrowding and access block pose great dangers to patient safety [1]. Significant contributions from patient flow research include the identification of strategies to reduce demand for emergency services, improve hospitalwide management of system capacity, and expedite emergency throughput through process improvements such as patient streaming, point of care laboratory tests, fast-track treatment zones, and short stay observation units [1,2,6].

Throughout this research, emergency patient flow has been widely conceptualized using linear models that illustrate patient transitions into, through, and out of the emergency department [1,8]. However, *s*tudies that represent patient flow as a simplified sequential process may fail to account for the impact of the human agents who actively manage patient flow [9-12]. Research that does not adequately consider human factors has been criticized for poorly capturing the complexity of real-world systems, lacking generalizability across emergency departments, and failing to provide a deeper understanding of how and why patient flow interventions succeed or fail [4,6,13,14]. Emergency nurses are autonomous decision makers who exert active agency over care processes and have a demonstrated impact on patient flow outcomes [5,10,15]. Nevertheless, few scholars have investigated this aspect of nursing work [5,15].

To respond to this gap in knowledge, this paper is the second in a series that describes emergency nurse patient flow management. For the purposes of this research, “emergency nurses” describes registered nurses employed in hospital-based or freestanding emergency departments. The first paper described how emergency nurses conceptualize patient flow management as the balancing practice of optimizing patient care without exhausting department resources [16]. This balance is guided by the primary goal of patient safety [12,16]. Patient flow management encompasses five critical tasks: (1) information gathering, (2) continuous triage, (3) resource management, (4) throughput management, and (5) care oversight [16]. By engaging in these tasks, emergency nurses monitor fluctuating resources and patient care needs and aim to ethically allocate limited resources to the right patient. Emergency nurses also manage a tension between the desire to expedite patient throughput and the desire to provide care that comprehensively meets patient needs [16].

More specifically, nurses manage the flow of patients by making real-time, frontline decisions to shape patient care [12,16]. Nurses are often responsible for triaging patients and determining the order of bedding assignments, placing patients in appropriate treatment spaces, choosing which patients receive limited department resources such as cardiac monitors and specialized treatment rooms, and managing the size and acuity of nursing assignments [16-18]. Nurses also influence the timeliness and order of patients to receive treatment, diagnostic testing, inpatient bed assignment, and discharge or transfer [16-18]. Patient flow management is understood to be complex, dynamic, and cognitively demanding [6,12,15]. This paper adds to previous descriptions of emergency patient flow management by analyzing the factors that shape nursing work systems.

### Human Factors and the Systems Engineering Initiative for Patient Safety Model

A purposeful investigation of interactions between humans, processes, and their environment is needed to effectively design systems, promote worker well-being, and understand and improve worker performance [19]. However, human factors methods have been underutilized in emergency medicine, and the organizational, cultural, and interpersonal factors that impact emergency patient care have yet to be fully explored [20-22]. Barriers to applying human factors approaches within emergency medicine include a lack of resources within hospitals to conduct human factors research, policies that impede human factors experts from accessing clinical settings, and limited dissemination of published examples [20].

**Figure 1.**
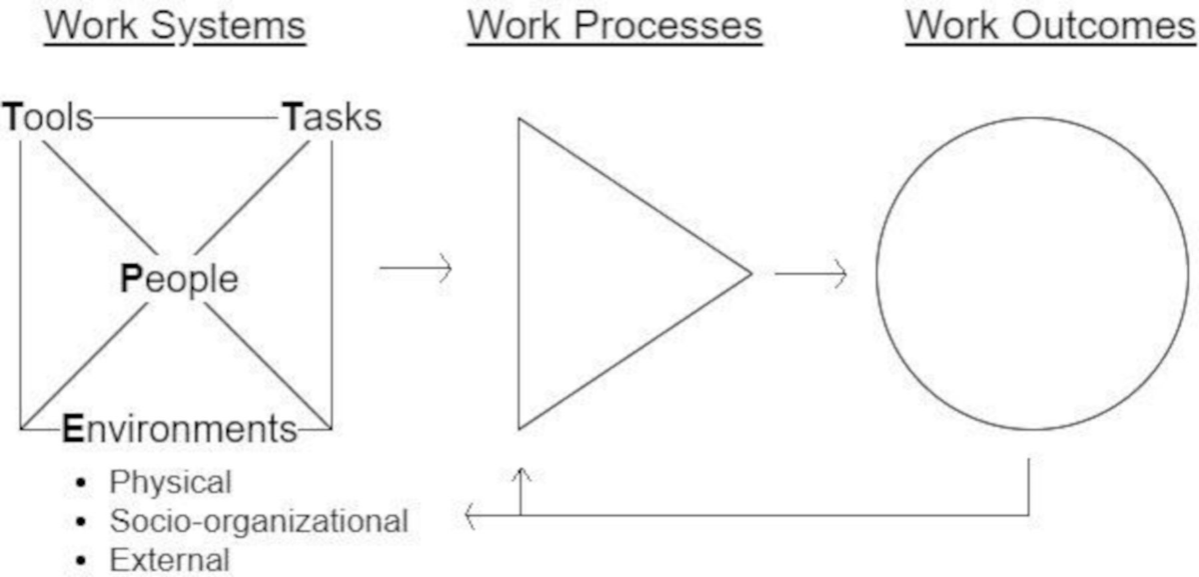
SEIPS 101 model by Holden and Carayon [26], published under Creative Commons Attribution-Noncommercial 4.0 International License [34]. SEIPS: Systems Engineering Initiative for Patient Safety.

The Systems Engineering Initiative for Patient Safety (SEIPS) model provides a human factors–based framework to investigate health care work [23]. The SEIPS model was first proposed in 2006 and has been refined several times, including the recently simplified SEIPS 101 model (Figure 1) [23-26]. SEIPS builds upon Donabedian’s classic System-Process-Outcomes model [27] to further explicate the elements of work systems, work processes, and work outcomes [23]. Within emergency health care research, this model has been successfully used to investigate physician documentation practices, physician disposition decision-making, and the transitions of older adult patients back home [28-30]. Within nursing, the SEIPS model has been used to better understand the work systems factors that impact nursing medication errors in critical care units, cardiac nursing workflow, and endotracheal cuff pressure management [31-33].

This manuscript focuses on the work system, which is the first element of the SEIPS model [26] (Figure 1). The work system comprises 6 interconnected components: people, tasks, tools, physical environment, socio-organizational environment, and the external environment. These elements interact to form the work processes that directly impact outcomes such as health care quality, safety, and worker well-being [23].

## Methods

### Study Design

This study employed constructivist and situational analysis grounded theory methodologies, as articulated by Kathy Charmaz and Adele Clarke. Each methodology involves inductive, qualitative analysis [35,36]. Constructivist grounded theory focuses on exploring social processes [36], while situational analysis supports analysis of the disparate, nonhuman elements that shape situations [35]. To minimize bias and preconceptions, grounded theorists may postpone formal literature reviews until after data collection and analysis [36]. Therefore, this study started with the broad question, “How do emergency nurses perform patient flow management?”

### Data Collection

Data collection strategies included individual interviews, focus groups, and observations across 4 emergency departments. Interview and focus group participants were recruited using purposeful and snowball sampling through email and social media platforms. Participation inclusion criteria were the following: English-speaking, over the age of 18 years, and registered nurses with at least 90 days of experience working in an emergency department. Initial interviews and focus groups lasted approximately 60 minutes and were guided by broad interview guides. Meetings were held remotely over Zoom and were audio/video recorded and transcribed with manual verification.

After initial rounds of focus groups and interviews, theoretical sampling was used to recruit existing participants for additional rounds of follow-up and think-aloud scenario interviews. Think-aloud scenario interviews [37] prompted participants to verbalize their patient flow management considerations when presented with a mock, simulated emergency department tracking board. These subsequent interviews were conducted individually, lasted between 30‐60 minutes, and were used to support and further develop coding categories. In all, a total of 5 focus groups and 24 interviews were conducted across 27 participants.

Participant observations were concurrently conducted at 4 emergency departments in the northeastern United States. Departments had varying community settings, sizes, trauma designations, and annual patient visit volumes. The researcher recorded handwritten narrative field notes to describe the departments and staff behavior. Field notes also described brief interactions with staff that were used to clarify their actions and decision-making strategies. Observations were conducted in 4-hour blocks at variable times throughout a 24-hour period, for a total of 64 hours of observation.

### Data Analysis

Data analysis occurred simultaneously with data collection and continued until saturation was reached. Data analysis relied on coding in NVivo 12 Plus (Lumivero) and constant comparative analysis. Line-by-line in vivo and gerund coding were used for initial coding [36]. As data analysis progressed, incident-by-incident coding was also used to code larger segments of data [36]. Constant comparative analysis is a method of qualitative analysis where coding of incidents and categories are compared to previous analysis to clarify emerging themes, inform additional data collection, and generate a theory [38]. Memo-writing was used to investigate coding categories, clarify theory development, and prompt reflexivity. Data collection and analysis were performed by a researcher experienced in emergency nursing; thus, reflexivity was an active process of investigating assumptions and personal experiences, as well as reflecting upon potential biases [36].

After emergent themes were developed, a review of the literature was performed, and study findings were found to closely align with the SEIPS model (Table 1). Emergent themes were then recategorized according to the SEIPS framework. As Carayon et al state, the SEIPS model can be applied during data analysis even if it was not used to guide data collection [24].

**Table 1.** Emergent theme and corresponding SEIPS component.^a^

Grounded theory emergent theme	Corresponding SEIPS component
Tasks of patient flow management	Tasks
**Factors that impact patient flow management**	
Individual nursing factors	Person factors
Departmental factors (structural: resources, technology, physical layout; interpersonal: communication, staff roles and norms, department culture)	Technology and tools, and physical and socio-organizational environment factors
Interdepartmental factors	External environment factors

a SEIPS: Systems Engineering Initiative for Patient Safety.

### Trustworthiness

Strategies to increase study trustworthiness included prolonged engagement with data, negative case analysis, triangulation of participant samples and data collection strategies, and use of an audit trail. Three formal member checking interviews were performed and affirmed study findings. Study design, data analysis, and theory generation were informed by consultation with methodology and subject matter experts.

### Ethical Considerations

This study was approved by the institutional review boards of the participating health care system and the University of Massachusetts Amherst (number 017066‐00002). A Certificate of Confidentiality from the National Institutes of Health was obtained to increase participant confidentiality. Informed consent was obtained from focus group and interview participants using Qualtrics surveys. Observed participants were informed of the study using an information sheet and interactions were guided by an oral script with verbal confirmation of participation. Focus group and interview participants were compensated at a rate of US $35/hour using Amazon gift cards.

## Results

### Overview

**Figure 2.**
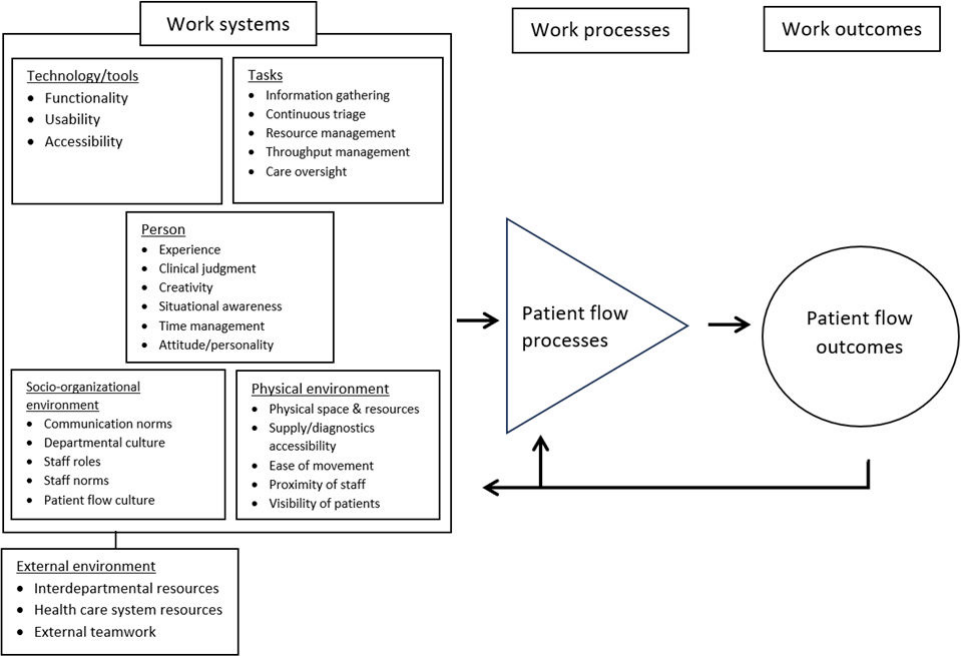
A SEIPS model of emergency nurse patient flow management. SEIPS: Systems Engineering Initiative for Patient Safety.

Study findings are categorized according to elements of the SEIPS work system: person, technology and tools, socio-organizational environment, physical environment, and external environment (Figure 2). Participant demographics, emergency department setting characteristics, and patient flow management tasks are described in detail elsewhere [16]. Table 2 summarizes supporting evidence.

**Table 2.** Summary of findings with supporting evidence.

Work system element, major theme, and minor theme			Supporting quotes
**Person**			
	***Experience***		
		Emergency department	It’s taking him a long time to adjust to this environment...in the ICU, “patients are there for so long, their plan of care is established.” (RN2) – Field Note 14
		Overcrowding experience	Community hospitals have a harder time dealing with stress influxes on patient resource management, while larger hospitals are used to being consistently stressed… and handle that patient flow management under stress a little bit better. (Int5)
		Experience in specialized flow roles	When you’re able to take those experiences, it opens your eyes up to the bigger picture…of patient flow. (Int11)
	Clinical judgment		It has to be somebody with...that has a good core knowledge as a base. (Int6)
	Creativity		Part of the charge nurse’s job is to be really creative… you have to be innovative. (FG5)
	***Time management***		
		Correct prioritization of tasks	So I guess prioritizing…having a great awareness of prioritizing what is important at the time. (Int7)
		Ability to multitask	I think that’s where a lot of nurses really get caught up, is they don’t have the ability to effectively multitask and remember the 7 different directions that they need to go at once. (Int10)
	Situational awareness		Our charge nurses, I think of them as up in the balcony, watching the orchestra play. They’re imperative to the functioning of the team, but they’ve gotta be up a level so that they can actually see what’s going on, and so they’ve got that complete view that isn’t skewed because they’re, you know, sinking in the quicksand with everybody else. (Int1)
	***Attitude***		
		Sense of accountability to department	The nurses are wrapped up in their own little section, and they don’t want more because they’re overwhelmed. (FG3)
		Degree of motivation	And then you have bedside nurses who will sit on patients, sit on orders, to hold on to as many patients as they can, so that they don’t have openings and flow. (FG5)
		Level of burnout	I’ve never seen nurses this exhausted, physically and emotionally. (Int6)
	***Personality***		
		Compassion for patients and colleagues	I think they’re good people… it’s definitely a personality thing. They’re friendlier, more socially aware of other people’s struggles, and they care about it. I think other nurses don’t give a shit. (Int9)
		Adaptability	There’s some people that they’re like ‘Chicken Little the sky is falling’ all the time, and it’s like, ‘Take a deep breath. It’s gonna be okay.’ (Int2)
**Technology and tools**			
	Functionality		We all have the computers on wheels and they’re just all over the place…and you know, of course, half of them don’t work. (Int8)
	Usability		I was trying to login so I could get report, and it pops up, ’Your patient is overdue for a bath.’ It’s like, ‘Why, why?!’...and you can’t get out of the screen unless you chart something, and if you chart “not responsible,” it tries to prompt you as to why?... and then it’s, you’re clicking out of the boxes... it’s alarm fatigue, but for the charting side. It’s charting fatigue. (Int15)
	Information accessibility		So when an ambulance comes in, I don’t like to be like, ‘Oh, hold on let me change my screen.’ I just wanna know, visually, like what I have available, and who has what, and that kind of thing, at all times. (Int7)
**Physical environment**			
	Physical capacity and resources		There’s just no space, no room. Even if we try to bring them back, there’s just nowhere to put them. (FG12)
	Access to supplies and diagnostics		Our isolation room has a little anteroom…we were kind of using it for storage, and we would just shove stuff in there, all kinds of stuff. And a lot of equipment has to be plugged in all the time, so their solution was to mount an extension cord strip type thing to the wall… so all that equipment is just right there, to get to sterile gloves, I have to move things out of the way. (Int8)
	Ease of movement		There wasn’t even a hallway space to put the patient in. It was like, ‘Okay, I have 14 inches here in a hallway.’ Everybody just turned sideways to go around this trauma patient. (FG11)
	Proximity of staff		Just having everybody kind of close to the nurses’ station just really helps communication. (Int7)
	Visibility of patients		The worst setup that we did was fast-track. They were made all private rooms, and you couldn’t see the patients ‘cause they were behind a closed door. (Int6)
**Socio-organizational environment**			
	Communication norms		If you have [staff that] don’t openly communicate, you are going to have significant delays in your patient flow management. (Int5)
	Staff roles		There was no flow coordinator. I had never heard of that until my friend was talking about their flow coordinator, I’m like, ‘Who is that?’ (Int15)
	***Staff norms***		
		Role flexibility	If I’m lead that day, like, I have my plan… I have my system. And if people do try to help …I think it adds to the chaos. (Int7)
		Role preference	That’s a bad job by the charge person, because you’ve got all your buddies doing all the fun jobs for the day, and then everyone else has to do the other ones. And if you don’t think they notice that, you’re out of your mind. So that’s a cultural problem that will slow things down. (Int9)
		Role hierarchy	Good patient flow has to be somebody who’s willing to not sit in the White Tower… or ‘I’m gonna sit in the pod chair and get ‘charge butt’ and never move.’ You have to move. (Int3)
	***Departmental culture***		
		Teamwork and camaraderie	They would step over your quivering body on the floor to get to where they’re going, instead of picking you up or helping you. (Int9)
		Respect between providers and nurses	If they walk up to a doctor, whether it’s a third or fourth year resident or an attending, and they say ‘You need to come right now and see this patient.’ That doctor will stop what they’re doing and they will go see their patient. (Int6)
		Relationship between staff and administration	It’s so frustrating, ‘cause this is the 6th hospital I’ve worked in, and they’re all the same. They’re greedy, greedy corporations and they don’t care about patients. (RN6)
		Capacity for change	RN2 tells me some of the older nurses are “stuck in the old ways” when they only saw 5 patients in their whole shift and they’ve had trouble adjusting. — Field Note 11
	***Patient flow culture***		
		Perception of provider incentives	I’ll have a patient come in from the waiting room and they’re already admitted. And it’s for bullshit reasons…They need to look at what they’re admitting and send more people home. (RN7)
		Provider-driven versus nurse-driven flow	So now, it’s interesting for me to come back and see this clash between what the doctors are used to. They’re used to nurses running things, and the nurses are used to doctors running things. (Int12)
		Role of patient flow metrics	Like door-to-doc time less than 20 minutes, discharge time less than 7 minutes...it was engraved in everybody’s head. As soon as that manager left—we just got a new manager—and there’s no real guidance for this new manager. (Int7)
**External environment**			
	Interdepartmental resources		That’s our biggest pushback... we’ve had a CT scanner down and they’re understaffed right now, and we had a 4-hour delay in CTs yesterday. (Int2)
	Health care system resources		There’s this backlog… when patients haven’t been efficiently discharged from upstairs, and there’s a delay to the folks who have been admitted. And then, obviously, it’s probably the same thing that’s happening in the bigger hospitals, so there’s no transfers available. (FG5)
	External teamwork		But the house supervisor will come and help—sometimes they’ll send…a nurse over here, who comes in kicking and screaming. (Int8)

### Person

Emergency patient flow management relies on nurses’ individual characteristics, including their level of experience, cognitive skills, and personal attributes. In addition to overall years of nursing experience, participants emphasized a need for experience working specifically in emergency settings. Emergency departments have their own unique rhythm and pace of care. In observations, this was exemplified by a senior intensive care unit (ICU) nurse who struggled with the speed and turnover of emergency patients.

RN2 has significant ICU experience, including 8 years working in a MICU, SICU, NICU [medical, surgical, neonatal ICU]. “It’s a totally different environment and flow than I’m used to.”

[Field Note 14]

Emergency department experience is also needed to gain knowledge of emergency medical treatment and care processes. Strong patient assessment skills, critical thinking, and knowledge of emergency patient care are perceived as the “core knowledge” (Int6) of emergency patient flow management. Notably, patient flow management is viewed as work that nurses learn after they master these fundamentals of emergency nursing. As one participant stated, “a new nurse is just trying not to kill a patient. And not miss anything” (RN3).

Grounded in clinical judgment, patient flow management also relies on nurses’ creativity, time management, and situational awareness. To successfully manage flow, nurses must think on their feet and creatively problem-solve in real time, while simultaneously managing multiple, urgent patient care priorities. This creativity is fostered by experience in overcrowded settings, where nurses learn to “think outside the box” (Int1) and develop a repertoire of innovative strategies.

Above all, participants emphasized the need for situational awareness. Situational awareness was described as the ability to maintain a holistic perspective of fluctuating patients, care needs, staff, and resources. Nurses with strong situational awareness are knowledgeable about the entire emergency department within its wider contexts of the hospital, emergency medical system, and surrounding health care system.

*So you need to be able to look at the big picture, not just your one team of patients, or even your one ER. You have to be able to look and see globally what’s going on.*

[Int4]

The cognitive skills required to manage patient flow are strengthened through experience in specialized flow roles. Nurses in lead, float, flow coordinator, journey navigator, resource, or charge roles are immersed in the work of managing patient flow across an entire pod, zone, or department. As one participant described, these roles “open your eyes up to the bigger picture of patient flow” (Int11) and provide nurses with a deeper knowledge of the policies, organizational procedures, and internal politics of their institution.

Patient flow management is also impacted by nurses’ attitudes and personalities. Nurses engage more in patient flow management when they have a sense of accountability to the wider department. Nurses who focus more narrowly on their own patients often lack an awareness of the department’s patient volume, the number of waiting patients, or of coworkers struggling with heavy assignments.

They’re just focused on their assignment…they work on their own pace and are in their own bubble. They're not hustling as much as the charge nurse would be, or somebody who’s trying to make room for other folks.

[FG5]

Nurses’ engagement in patient flow management also varies according to their level of motivation. Many nurses demonstrate a sense of urgency to expedite patient throughput. Other nurses are described as “unmotivated, complacent, or lazy.” Burnout was seen as an especially common barrier to patient flow management engagement. Participants reported widespread physical, mental, and emotional exhaustion among emergency staff. For disengaged nurses, expediting patient flow is undesirable because it leads to new patients and additional work. As one participant summarized, “the more you kick in quicksand, the faster you sink” (FG3).

*Well, the faster I get this out, the faster you're just gonna give me something new, and this never gonna end, so why should I hurry?*

[FG4]

Regarding personality, patient flow management is impacted by nurses’ level of compassion and adaptability. Nurses are perceived as more highly engaged when they hold deep compassion for their patients and colleagues that drives a desire to help and provide better care. Effective patient flow management also relies on the ability to remain calm and level-headed under pressure.

None of my nurses really spin, you know, they don't get caught up and start freaking out about things. My team is so tight…we just don't get that excited over stuff.

[Int10]

To summarize the personal factors, patient flow management appears to be facilitated by highly engaged nurses who are experienced in emergency department care, have deep clinical and institutional knowledge, and have the cognitive capacity to perform creative, complex, dynamic work.

### Technology and Tools

Technology was found to significantly shape emergency nurse patient flow management. Access to Pyxis medication dispensers, portable computers, pneumatic tube systems, portable communication devices, and online applications that provide pharmacy or education support were observed to impact nurse workflows. Health information systems, including emergency tracking boards, electronic medical records, bed tracking systems, and ambulance tracking systems, appear to be especially consequential to patient flow management because they shape emergency nurses’ ability to gather information about department resources and patient care.

When describing technology, participants reported concerns with functionality, usability, and information accessibility. Inconsistent access to functioning technology is a common and time-consuming barrier to patient flow management. Participants frequently dealt with failures and breakdowns such as broken medication scanners, computer downtimes, stalled pneumatic tube systems, and challenges with portable communication devices.

*Nothing is working...yawn...the computers aren’t working*. (RN3)

[Field Note 11]

Participants also struggled with technology usability, often lacking a full understanding of how to operate and troubleshoot devices or make sense of presented information. Emergency nurses heavily rely on emergency tracking boards, which present a departmentwide overview of patient assignments and care progress. Multiple participants acknowledged uncertainty in the meaning of the icons, symbols, and colors displayed on the emergency tracking board.

*… the stars indicate if an order is late by 15 minutes or 30 minutes. They both laugh, “See? I didn’t even know that.” (RN3)*

[Field Note 8]

Although some participants reported satisfaction with their health information systems, many described information platforms that were overwhelming, contained burdensome alert functions, and had poorly designed interfaces.

I ask about their experience using [product]. “It’s overwhelming,” “Way too much on the eyes. I don’t know how pod leads can look at this board.” (RN10)

[Field Note 8]

Last, information accessibility appears to be impacted by the number of different health information systems required to access patient information and the transparency between different systems. Participants reported outdated programs, the need to navigate several different interfaces, and time-consuming workarounds.

*To look up the actual lab numbers you have to go to [a] different screen, to look up radiology results you have to go to [a] different program, and so there’s a lot of minimizing, you know, getting to this screen, or that screen, or this screen, or that screen, to figure out what needs to be done.*

[Int8]

### Socio-Organizational Environment

Patient flow management is impacted by the social and organizational characteristics that influence how nurses communicate and work together. First, participants emphasized the importance of clear, closed-loop, and frequent communication to keep one another apprised of department resources and patient care. Observations revealed that, in practice, nurses often rely on indirect strategies, such as writing updates on the emergency tracking board, to communicate changes in patient locations. Communication failures were especially common during handover with prehospital personnel, during bedding assignments, and when calling patient report to inpatient units.

*“Do you have that guy?”* (RN5) *“Oh, so I probably have that guy. I don’t know, I just came out and my name was on them. Alright, I guess I’ll go see him.”* (RN4) *The patient they’re talking about has been in the department for 2 hours.*

[Field Note 3]

Patient flow management is also shaped by the culture of emergency staff, providers, and administration. Departments with high levels of teamwork and camaraderie are able to work together toward common patient flow goals. Participants described respect between providers and nurses as the foundation for patient safety and optimized flow. Without a sense of mutual respect, nurses report a reluctance to ask questions, raise concerns, and share their opinions. Whereas nurses praised departmental cultures where staff and providers were a unified team, participants often expressed a disdain or distrust of hospital administrators. Nurses’ suspicion of administrators’ intentions can exacerbate a reluctance to embrace change or engage in patient flow improvement initiatives.

With this model and this process that they want to try—that was directed by our medical director, who is not a nurse, and he doesn't push for us to get these patients out like he should, or stand up to the people with the dollars and the pulling of strings.

[Int14]

Staff roles were found to vary widely between departments, encompassing a broad array of role titles and job functions. Responsibility for patient flow management decisions may be held by staff nurses, charge nurses, or by a variety of specialized flow roles such as flow coordinators, triage nurses, navigator nurses, pivot nurses, pod leads, float nurses, streamer nurses, or bed czars. Individual roles fluctuate according to staffing levels, such that one staff member may have to take on the duties of several roles during periods of poor staffing. Patient flow management is also shaped by the presence of other staff and provider roles, such as technicians, medical assistants, paramedics, orderlies, transporters, and medical residents.

*And then the roles within the department, your typical staff nurse—clinical nurse, but we also have a resource nurse, which is just a charge nurse. We also have a flow nurse and then triage nurses as well.*

[FG17]

In addition to differing staff role titles and functions, departments vary in their role norms. Some departments demonstrate high levels of flexibility in responsibility for patient flow management decisions, while others have more rigid role expectations. For example, in some departments, the responsibilities of answering the ambulance radio, assigning patients to emergency rooms, or shuffling patient locations are strictly held by a charge or flow coordinator nurse. In other departments, nurses in bedside roles readily take on these tasks when they see an opportunity to help. Departmental norms further impact the desirability of certain roles and role hierarchy. High degrees of hierarchy that create distance between the charge nurse or flow coordinators and the patients’ bedside may impede that nurse’s familiarity with direct patient care and create animosity between staff.

*I feel like when some people level-up they forget how it is to be in an assignment. And then they’re in an assignment and they’re drowning and they’re asking you for your help.*

[RN4]

Role norms also impact whether patient flow management is perceived as primarily provider- or nurse-driven. Departments perceived to be more “doctor-driven” were described as those where the providers more closely monitor the waiting room patients and voice their opinions about who should be assigned emergency beds, and where providers delegate frequently and engage less in collaborative decision-making.

*RN2 said that post-COVID, with all the newer nurses, that patient care is more “micromanaged” by the doctors. “It never used to be like that.” (RN2)*

[Field Note 8]

Finally, patient flow management is influenced by the incentives provided to physicians and nurses. Participants criticized the perception that providers may be incentivized to empty the waiting room, perform excessive diagnostic imaging, and admit high proportions of emergency patients as especially impactful on patient flow management.

Our doctors… hate waiting room times. So at (hospital), what I appreciated was it was okay to have a waiting room. That’s what the waiting room is for.

[FG17]

Emergency departments vary in the extent to which management has established clear incentives to meet patient flow metric goals. These metric goals might include specifications for the length of time that patients should wait to be assessed or transported to an assigned bed. Nurses differ in their opinions of these patient flow standards. Although some participants felt that strict timing expectations were helpful to increase staff engagement, others felt that they compromised nursing judgment and patient care.

It made it harder to be really thorough. Like you’d think ‘Hmm, I can either do a really thorough assessment, or I can meet my time.’

[RN2]

### Physical Environment

Participants perceive access to resources to be the single most important factor influencing patient flow management. Patient flow management is impeded by insufficient beds, rooms, equipment and supplies, and inadequate staffing.

The halls are narrow, especially when we have the hallway beds in there. So, you know, when you're pushing a gurney through there, you're kind of having to wiggle a little bit, make sure you don't hit somebody.

[Int8]

In addition to its physical capacity, the layout of a department impacts nurses’ access to supplies and diagnostic testing, physical movement, the proximity of staff, and visibility of patients. These characteristics impact the efficiency and ease of emergency nurses’ work. In observations, departments were found to be cluttered, cramped, disorganized, and lacking sufficient capacity for patients, medical equipment, and supplies.

*So layout is important…because there are rooms where you're kind of isolated, and it’s hard because they don’t hear the doctors talking, or updates on patients, so that has an impact on patient flow.*

[Int7]

### External Environment

Last, emergency patient flow management is shaped by factors external to the work system of the department. Interdepartmental resources—including staffing levels of diagnostic departments, environmental services, transport services, and inpatient units—impact the efficiency of patient care and movement out of the department. Participants also acutely felt the consequences of working within an overburdened health care system that limits their ability to successfully transfer and discharge patients. Nurses emphasized a frustration with the lack of adequate ambulance transportation.

Our EMS service’s so short-staffed, they can hardly handle the 9-1-1 calls, let alone handling the transports out. Like, how do we get these people out?

[FG4]

Teamwork between the emergency department and other hospital departments impacts the ability to coordinate and advance patient care processes. Tension and pushback from inpatient floors during reports from the emergency department to floors is a common experience. Participants reported that inpatient nurses are often unavailable to take report, reluctant to accept patients, and engage in delaying tactics. This pushback from the floor results in challenges transporting emergency patients out of the department.

*It’s been like hand-to-hand combat with the floors, trying to get patients upstairs.*

[Int10]

## Discussion

### Principal Findings

This is the first paper, to our knowledge, to comprehensively describe the work system of emergency nurse patient flow management. Using this approach, we have framed patient flow processes around the work processes of nurses rather than sequential patient transitions. The articulation of 5 discrete patient flow management tasks is described more thoroughly in the first paper in this series [16]. Conceptualizing patient flow management as a balance of tasks offers a new framework for improving patient flow by supporting the nursing work of information gathering, continuous triage, resource management, throughput management, and care oversight.

The SEIPS model places a “person” at the heart of the work system. This human-centric approach theoretically links nurses’ individual characteristics to patient flow outcomes. Study results propose that patient flow outcomes are influenced by emergency nurses’ attitude, personality, experience level, and cognitive abilities. Collectively, these findings emphasize the importance of strengthening nurse training, retention, and support. Developing nurse patient flow management training has been proposed as a potentially cost-effective approach to improve patient flow [15]. The current lack of training may be due, in part, to a limited understanding of needed skills and traits [39]. Nursing education also predominately focuses on individual, patient-centered care rather than collective decision-making across multiple patients [16,40]. This study has clarified that clinical judgment, time management, and situational awareness are essential for patient flow management and should be a priority for training efforts. In addition to training support, findings suggest that hospitals may consider investing in nurse retention and well-being as a patient flow intervention.

Patient flow management depends on several structural factors, including department technology, physical capacity, layout, and internal and external resources. These elements impact the speed and ease of nursing work, including the ability to gather information, access needed supplies, collaborate with peers, and visualize patients. Broadly, participants described emergency departments as ill-suited to support the work of patient flow management. Nurses criticized the accessibility, functionality, and usability of health information systems, and physical work environments were found to be cramped, cluttered, and lacking adequate supply and staffing resources. Although the physical capabilities and resource levels of emergency departments are often challenging to change, there is potential to better support patient flow managers by redesigning health information systems [41]. Notably, a recent systematic review found that, despite their importance to patient flow, how and why health information systems impact patient flow processes remain poorly understood [42], re-emphasizing the need to better understand emergency nursing work processes.

Finally, patient flow management is shaped by social and organizational factors. Although the importance of departmental communication and culture to promote patient flow are apparent, the variability of staff roles, staff norms, and patient flow incentives are noteworthy findings. Patient flow management roles, norms, and incentives were found to be highly inconsistent, context dependent, and largely informal. Staff roles and norms lack uniformity between departments and fluctuate as staffing levels, individual staff members’ preferences, and levels of expertise change. Departments also vary in the degree to which patient flow management is perceived to be doctor- or nurse-driven, and these expectations are unwritten and obscure. Unclear responsibility for patient flow management decision-making is further complicated in departments with high role flexibility, where flow decisions are made on an ad hoc basis by available or nearby nurses. Other scholars have noted this ambiguity and blurring of emergency department flow management roles and have called for greater clarity in staff responsibilities [43-45].

Further, findings suggest a great disparity between departments’ enforcement of patient flow metric expectations. Some nurse participants described working in environments with highly stringent timing guidelines, while other nurses were unsure if their hospitals had any timing expectations at all. The impact of these patient flow incentives is unclear. Scholars have recognized an inherent tension between quality and speed of care in the work of patient flow management [9,16,45], but research is needed to understand how emergency nurses manage this balance. Overall, strategies to clarify and promote consistency between patient flow management roles, norms, and incentives may benefit emergency nursing work.

### Implications and Applications

This paper has addressed a gap in research describing emergency nurse patient flow management. We have identified numerous work system elements that shape emergency nursing work. These work system elements are theoretically linked to patient flow outcomes, but more research is needed to verify and measure their impact. The presented SEIPS model should therefore serve as a guiding framework for future studies that investigate the facilitators, barriers, and motivators to emergency nursing patient flow management.

This SEIPS work system analysis further demonstrates the complexity and difficulty of patient flow management. Patient flow researchers and health care administrators should embrace the study of human factors to support health care delivery. Increasing scholarly attention to the work of emergency nurses may offer new strategies to improve patient flow.

### Impact Statement

The work of emergency nurse patient flow management has been poorly described. This nursing research study has used a human factors model to analyze the work system of emergency patient flow management. Study findings offer a theoretical framework to further investigate the impact of emergency nursing work on patient flow outcomes and identify novel patient flow solutions.

### Conclusion

The SEIPS model has been applied in many health care projects and sectors to investigate care delivery [24]. Findings from this inductive, qualitative study provide further empirical support for the validity and usefulness of the SEIPS model. The unique contribution of this paper is the integration of the SEIPS model with the expertise of emergency nurses to describe their work system. There are many opportunities to better support emergency nurses in the complex, dynamic work of patient flow management.

## Abbreviations

ICU: intensive care unit
SEIPS: Systems Engineering Initiative for Patient Safety

## References

[1] Javidan A, Hansen K, Higginson I (2020). White paper from the emergency department crowding and access block task force. https://assets.nationbuilder.com/ifem/pages/270/attachments/original/1650595379/ED-Crowding-and-Access-Block-Report-Final-June-30-2020.pdf?1650595379.

[2] Rutherford PA, Anderson A, Kotagal UR (2020). Achieving hospital-wide patient flow. https://www.ihi.org/sites/default/files/IHIAchievingHospitalWidePatientFlowWhitePaper.pdf.

[3] Benjamin E, Jacelon C (2022). An analysis of the concept of patient flow management. Nurs Forum.

[4] Saghafian S, Austin G, Traub SJ (2015). Operations research/management contributions to emergency department patient flow optimization: review and research prospects. IIE Trans Healthc Syst Eng.

[5] Hybinette K, Praetorius G, Ekstedt M, Pukk Härenstam K (2023). Exploring patient flow management through a lens of cognitive systems engineering. Ergonomics.

[6] De Freitas L, Goodacre S, O’Hara R, Thokala P, Hariharan S (2018). Interventions to improve patient flow in emergency departments: an umbrella review. Emerg Med J.

[7] Hall RW (2013). Patient Flow: Reducing Delay in Healthcare Delivery.

[8] Asplin BR, Magid DJ, Rhodes KV, Solberg LI, Lurie N, Camargo CA (2003). A conceptual model of emergency department crowding. Ann Emerg Med.

[9] Nugus P, Braithwaite J (2010). The dynamic interaction of quality and efficiency in the emergency department: squaring the circle?. Soc Sci Med.

[10] Nugus P, Forero R, McCarthy S (2014). The emergency department “carousel”: an ethnographically-derived model of the dynamics of patient flow. Int Emerg Nurs.

[11] Nugus P, Holdgate A, Fry M, Forero R, McCarthy S, Braithwaite J (2011). Work pressure and patient flow management in the emergency department: findings from an ethnographic study. Acad Emerg Med.

[12] Benjamin E, Wolf LA (2022). “ Nurses are every bit of the flow:” emergency department nurses’ conceptualization of patient flow management. Nurs Forum.

[13] Bergs J, Vandijck D, Hoogmartens O (2016). Emergency department crowding: time to shift the paradigm from predicting and controlling to analysing and managing. Int Emerg Nurs.

[14] Mohiuddin S, Busby J, Savović J (2017). Patient flow within UK emergency departments: a systematic review of the use of computer simulation modelling methods. BMJ Open.

[15] Sharma S, Rafferty AM, Boiko O (2020). The role and contribution of nurses to patient flow management in acute hospitals: a systematic review of mixed methods studies. Int J Nurs Stud.

[16] Benjamin E (2024). The work of patient flow management: a grounded theory study of emergency nurses. Int Emerg Nurs.

[17] Wolf L, Delao A, Simon C, Clark P, Burchill CN (2024). Ensuring throughput: development and validation of charge nurse competencies for United States emergency care settings. J Emerg Nurs.

[18] Reay G, Rankin JA, Then KL (2016). Momentary fitting in a fluid environment: a grounded theory of triage nurse decision making. Int Emerg Nurs.

[19] Carayon P (2012). Handbook of Human Factors and Ergonomics in Health Care and Patient Safety.

[20] Hayden EM, Wong AH, Ackerman J (2018). Human factors and simulation in emergency medicine. Acad Emerg Med.

[21] Gifford R, van der Vaart T, Molleman E, van der Linden MC (2022). Working together in emergency care? How professional boundaries influence integration efforts and operational performance. IJOPM.

[22] Chang AM, Cohen DJ, Lin A (2018). Hospital strategies for reducing emergency department crowding: a mixed-methods study. Ann Emerg Med.

[23] Carayon P, Schoofs Hundt A, Karsh BT (2006). Work system design for patient safety: the SEIPS model. Qual Saf Health Care.

[24] Carayon P, Wetterneck TB, Rivera-Rodriguez AJ (2014). Human factors systems approach to healthcare quality and patient safety. Appl Ergon.

[25] Carayon P, Wooldridge A, Hoonakker P, Hundt AS, Kelly MM (2020). SEIPS 3.0: Human-centered design of the patient journey for patient safety. Appl Ergon.

[26] Holden RJ, Carayon P (2021). SEIPS 101 and seven simple SEIPS tools. BMJ Qual Saf.

[27] Donabedian A (1988). The quality of care. How can it be assessed?. JAMA.

[28] Fouquet SD, Fitzmaurice L, Chan YR, Palmer EM (2021). Doctors documenting: an ethnographic and informatics approach to understanding attending physician documentation in the pediatric emergency department. J Am Med Inform Assoc.

[29] Rutkowski RA, Scheer E, Carlson C (2023). A scoping review of work system elements that influence emergency department disposition decision-making. Hum Fact Healthc.

[30] Werner NE, Rutkowski R, Graske A (2020). Exploring SEIPS 2.0 as a model for analyzing care transitions across work systems. Appl Ergon.

[31] Kebapci A, Ozkaynak M (2022). Endotracheal tube cuff pressure management: an observational study guided by the SEIPS model. Dimens Crit Care Nurs.

[32] Danesh MK, Garosi E, Mazloumi A, Najafi S (2020). Identifying factors influencing cardiac care nurses’ work ability within the framework of the SEIPS model. Work.

[33] Frith KH (2013). Medication errors in the intensive care unit: literature review using the SEIPS model. AACN Adv Crit Care.

[34] Attribution-noncommercial 4.0 international. Creative Commons.

[35] Clarke AE (2005). Situational Analysis: Grounded Theory After the Postmodern Turn.

[36] Charmaz K (2006). Constructing Grounded Theory: A Practical Guide Through Qualitative Analysis.

[37] Fonteyn M, Fisher A (1995). Use of think aloud method to study nurses’ reasoning and decision making in clinical practice settings. J Neurosci Nurs.

[38] Glaser BG, Anselm LS (1967). The Discovery of Grounded Theory: Strategies for Qualitative Research.

[39] Young C, Patey C, Norman P (2022). Identifying relevant topics and training methods for emergency department flow training. CJEM.

[40] Hossain F, Clatty A (2021). Self-care strategies in response to nurses’ moral injury during COVID-19 pandemic. Nurs Ethics.

[41] Almasi S, Rabiei R, Moghaddasi H, Vahidi-Asl M (2021). Emergency department quality dashboard; a systematic review of performance indicators, functionalities, and challenges. Arch Acad Emerg Med.

[42] Nguyen Q, Wybrow M, Burstein F, Taylor D, Enticott J (2022). Understanding the impacts of health information systems on patient flow management: a systematic review across several decades of research. PLoS ONE.

[43] Pryce A, Unwin M, Kinsman L, McCann D (2021). Delayed flow is a risk to patient safety: a mixed method analysis of emergency department patient flow. Int Emerg Nurs.

[44] Wise S, Duffield C, Fry M, Roche M (2022). Nurses’ role in accomplishing interprofessional coordination: lessons in “almost managing” an emergency department team. J Nurs Manag.

[45] Boiko O, Edwards M, Zschaler S, Miles S, Rafferty AM (2021). Interprofessional barriers in patient flow management: an interview study of the views of emergency department staff involved in patient admissions. J Interprof Care.

